# Comparative Study on the Surface Remelting of Mo-Si-B Alloys with Laser and Electron Beam

**DOI:** 10.3390/ma15186223

**Published:** 2022-09-07

**Authors:** Qiuliang Li, Cheng Wang, Zhuoyue Li, Yi Qu, Xiangrong Li

**Affiliations:** Fundamentals Department, Air Force Engineering University, Xi’an 710038, China

**Keywords:** dendrites, electron beam remelting, laser remelting, microstructure, Mo-12Si-8.5B alloy

## Abstract

The Mo-12Si-8.5B alloy was surface-remelted by laser and electron beam, and the microstructure of its melt pool and substrate regions were analyzed by scanning electron microscopy (SEM), X-ray diffraction (XRD), and energy spectrometry (EDS) techniques. It was found that the composition of the surface phases in the Mo-12Si-8.5B alloy did not change by the high-energy beam surface remelting process, but the microstructure of the molten pool region was significantly different from that of the substrate region, and its phase distribution was more uniform. Dendrites appeared on the surface of the material under the action of both processes, and the Si- and B-rich phases were mainly gathered in the interdendritic region. In the melt pool of the laser-remelted specimens, the α-Mo phase was continuously distributed with an average dendrite length of 70 µm, while the α-Mo phase distribution in the melt pool of the electron beam remelted specimens were relatively concentrated, with a larger dendrite size and an average dendrite length of 120 µm. The dendrite size in the melt pool of the laser remelted material was smaller, and the distribution of the elements was relatively uniform. Using a laser beam as the heat source was more favorable for the next step of the additive manufacturing of the core parts of hypersonic vehicles.

## 1. Introduction

With the development of aerospace technology and the increase in industrial production capacity, pre-turbine gas temperatures and the requirements for engine blade materials have increased [1,2]. Due to the limitation of its melting point, nickel-based superalloys cannot meet the urgent demand for increasing the performance of aero-engines, so other types of high-temperature materials need to be developed [3]. Mo-Si-B alloys are considered important candidates as high-temperature structural materials for next-generation aero-engines and hypersonic vehicles because of their extremely high melting point (above 2000 °C), excellent high-temperature strength, good high-temperature oxidation resistance and creep resistance [4]. Mo-Si-B alloys can increase the pre-turbine gas temperatures of aero-engines by 300–400 °C, greatly improving the maximum thrust and efficiency of aero-engines [5,6]. Currently, Mo-Si-B alloys are mainly prepared by arc melting and powder metallurgy [7,8], but the alloy structure obtained by arc melting often has serious elemental segregations that Si elements strongly segregate to dislocations and grain boundaries, which adversely affects the mechanical properties of the alloy [9,10]. The alloy structure obtained by powder metallurgy has light elemental segregation, just as Si elements have lighter segregations at dislocations and grain boundaries [9,10,11], but it is difficult to perform three-dimensional moldings of complex structures and is not suitable for the manufacture of aero-engine parts. However, the high-energy beam (laser beam, electron beam) additive manufacturing technology with a high temperature gradient (up to 10^7^ K/s between substrate and melt pool) and high cooling rate provides a new way to realize the three-dimensional forming of Mo-Si-B alloys. The high-energy beam surface remelting experiment can be seen as a metal additive manufacturing process with zero metallic powder volume.

Makineni et al. have conducted surface remelting of Mo-Si-B alloys and found that the remelted microstructures have more excellent oxidation resistance [12] and fracture toughness [13], indicating the great advantages of additive manufacturing technology for Mo-Si-B microstructures and property modulation. However, Mo-Si-B alloys have high melting points, high brittleness, and very poor welding properties, so achieving additive manufacturing of Mo-Si-B alloys still faces serious challenges. The first successful laser cladding fabrication of Mo-Si-B alloys was achieved by Schmelzer et al. [14], who obtained a crack-free cladding layer of approximately 3 mm in length by induction heating of the substrate at 600 °C. The microhardness of the cladding layer was comparable to that of the arc-melted alloy. Zhou et al. [15,16] also successfully used the laser-selective melting technique for the three-dimensional forming of ball-milled Mo-Si-B-Ti-C alloys, and due to the rapid solidification process of additive manufacturing, the final tissue formed had fine grain size and uniform distribution of TiC nanoparticles, but its microhardness was low compared with that of cast alloys of the same composition due to the microcracks existing inside the material. Fichtner et al. [17] have explored Mo-Si-B alloys by laser powder bed fusion and developed suitable process parameters for the generation of crack-free samples. Higashi et al. [18] have conducted Selective Laser Melting of Mo-Si-B alloys and found that rapid solidification via Selective Laser Melting (SLM) resulted in the refinement of microstructure and the formation of a supersaturated Mo_ss_ phase. At present, the research on the additive manufacturing process of Mo-Si-B alloys is still in the exploratory stage; there are few related studies, and there are still great challenges to forming Mo-Si-B alloy components using additive manufacturing technology.

The heat source types were considered a very important influencing factor in the formation of the melt pool microstructure [13,17]. However, the contrastive studies between laser beam remelting and electron beam remelting on the Mo-Si-B alloy are rare [13,14,15,16,17,18]. In this paper, two processes, laser remelting and electron beam remelting, are used to process the surface of the Mo-12Si-8.5B alloy. By comparing the molten pool with the substrate area and the organization of the molten pool under the two processes, we find out the changing pattern and promote the basic research on the application of Mo-Si-B alloys for preparing the hot end parts of aero-engines.

## 2. Materials and Methods

Powder mixtures with a nominal composition of Mo-12Si-8.5B (at.%) were prepared from Mo, Si, and B with 99.95 wt.%, 99.99 wt.% and 99.95 wt.% purity with an average particle size of 2.0–3.5 µm, 3.0–5.0 µm and 0.5–1.0 µm, respectively. The mixed powders were put into the planetary mill with a speed of 300 rpm and a powder-to-ball weight ratio of 1:10. Then, the base material used for the experiments was Mo-12Si-8.5B (at.%) alloy blocks obtained by discharge plasma sintering, numbered S1, S2, and S3, respectively, and the specific treatment process scheme is shown in Table 1.

The experiments were carried out by mechanically polishing the surface of Mo-12Si-8.5B alloy blocks first and then by surface processing according to the grouping. Before conducting the experiments, the Mo-12Si-8.5B alloy was pre-experimented to select the appropriate parameters. With the same line energy of the laser beam and electron beam, the process parameters with the best remelting effect on the sample surface were selected. The final process parameters were determined as follows: (1) The surface of the sample was remelted using a three-axis laser cladding device with a scanning speed of 900 mm/s and power of 800 W along the cross-section. (2) The surface of the sample was remelted using an electron beam with a beam current of 20 mA, accelerating voltage of 40 kV, and scanning speed of 900 mm/s along the cross-section. Laser remelting and electron beam remelting samples were cut using wire electro-discharge machining along the cross-section of the molten pool. The surface was electrolytically polished with a 1:7 volume ratio of sulfuric acid and alcohol solution and then etched with HF-HNO_3_-H_2_SO_4_ solution for 3 s. Phase identification was carried out by X-ray diffraction using a D8-Advance XRD instrument equipped with Cu Kα radiation (λ = 1.5406 Å) as a source operated at 40 kV and 25 mA, between 20 and 100 deg (2θ), at a step size (Δ2θ) of 0.03 deg and a counting time of 20 s per step. XRD parameters were chosen to obtain the best position and intensity of diffraction lines. Next, the surface morphology was analyzed using a JSM-6380 scanning electron microscope with its own energy spectrometer. The samples were mounted on a Scanning Electron Microscopy (SEM) carrier with adhesive conductive carbon tape. The SEM was operated at 25 kV to achieve the best signal-to-noise ratio and image resolution. Representative areas of the samples were analyzed and mapped for elemental distribution on the basis of the EDS data by QUANTAX ESPRIT microanalysis software. EDS mappings were taken from the selected regions operating with 25 kV of accelerating voltage. Porosity is measured with a fully automated mercury piezometer (MicroActive AutoPore V 9600, Micromeritics, Norcross, GA, USA). The Vickers hardness of the alloy was obtained by a microhardness tester (Wilson VH 3300, Norwood, MA, USA). The load and holding time were set to 196 N and 15 s, respectively; three points were selected for each area to be tested, and the average value was obtained. The appropriate parameters for the measurement were selected according to the study [13,14,15,16,17,18].

## 3. Results

### 3.1. Unprocessed Alloys

#### 3.1.1. XRD

**Figure 1:**
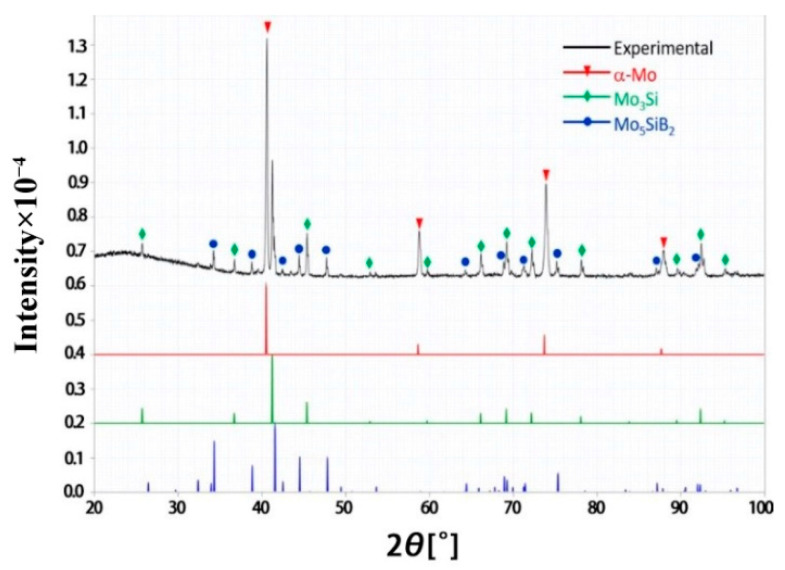
The XRD results of unprocessed Mo-12Si-8.5B alloys.

Figure 1 shows the surface X-ray diffraction spectra of Mo-12Si-8.5B specimens without any process treatment. It can be seen from the XRD results that the Mo-12Si-8.5B specimen obtained by discharge plasma sintering consists of three phases, α-Mo-Mo_3_Si-Mo_5_SiB_2_. This is a typical α-Mo-Mo_3_Si-Mo_5_SiB_2_ system, and the Mo-Si-B alloy has good toughness and ductility in the body-centered cubic molybdenum solid solution α-Mo. The continuous α-Mo solid solution can greatly improve the toughness and ductility of the Mo-Si-B alloy, while the intermediate phases, Mo_5_SiB_2_ and Mo_3_Si, have good high-temperature creep and oxidation resistance [19,20,21,22,23].

#### 3.1.2. Surface Microstructure of Unprocessed Specimens

**Figure 2:**
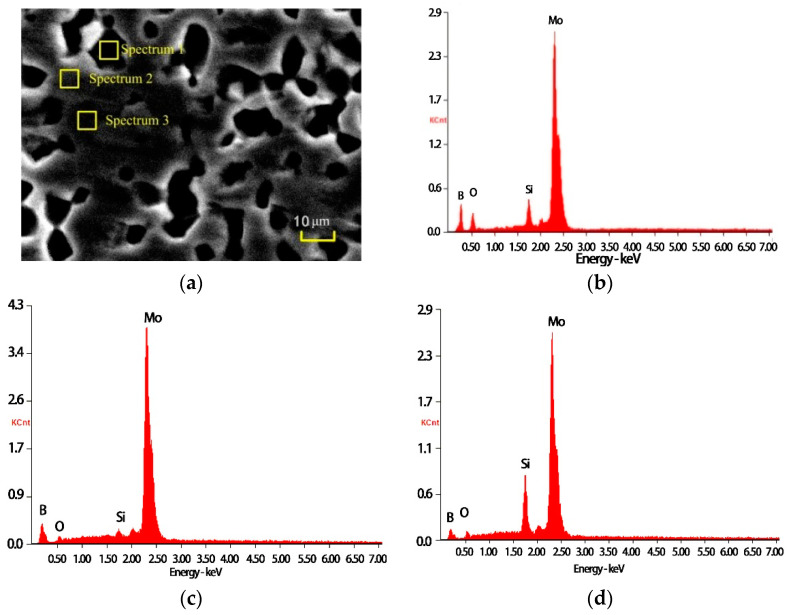

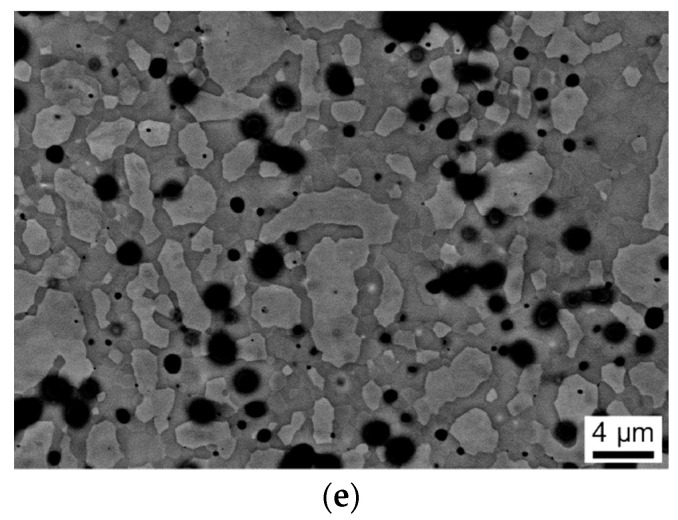
SEM micrograph and element composition of the unprocessed sample. (**a**) microstructure; (**b**) spectrum 1; (**c**) spectrum 2; (**d**) spectrum 3; (**e**) substrate.

The surface microstructure of the unprocessed specimen was analyzed by scanning electron microscopy and an energy spectrometer, and the results are shown in Figure 2. According to the XRD results and Refs. [7,22], the unprocessed specimen consists of three phases: α-Mo, Mo_3_Si, and Mo_5_SiB_2_, and the elemental contents and phase contrast of different phases have significant differences. Because the relative atomic mass of the B element is small, the content of the B element obtained by the energy spectrometer is not very accurate, so the distribution of each phase in Figure 2a can be determined by the ratio of Mo to Si elements. Figure 2b–d show the energy spectrometer results of the spectrum 1, spectrum 2 and spectrum 3 areas in Figure 2a, respectively. The ratio of Mo to Si in Figure 2b is about 5, which means that the corresponding spectrum 1 area in Figure 2a is a Mo_5_SiB_2_ phase, and the Mo_5_SiB_2_ phase is irregularly blocky and diffusely distributed in the substrate. The high content of Mo elements and very low content of Si elements in Figure 2c indicate that the relatively light spectrum 2 area in Figure 2a is the α-Mo phase. The ratio of Mo to Si elements in Figure 2b is about 3, indicating that the dark phase in the corresponding spectrum 2 area of Figure 2a is the Mo_3_Si phase. The α-Mo phase and Mo_3_Si complement each other and form the substrate together. The phase distribution on the surface of the Mo-12Si-8.5B material prepared by discharge plasma sintering is relatively uniform, but the phase distribution does not have a continuous character. Figure 2e shows the SEM image of the substrate region, and some holes are distributed on its surface.

**Figure 3:**
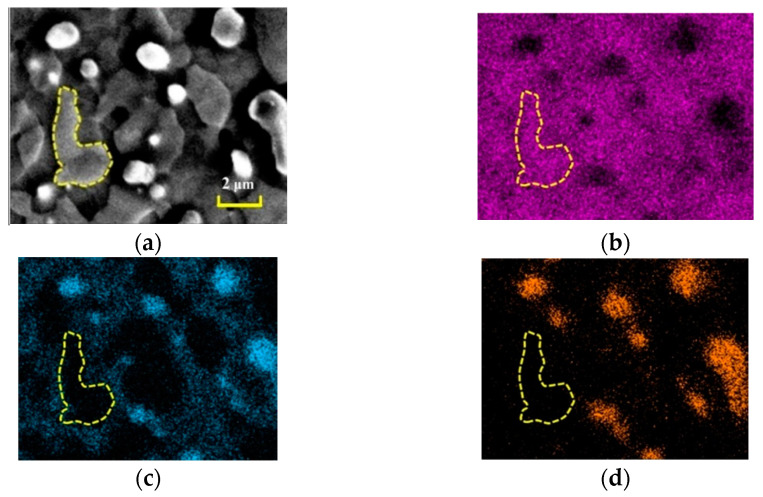
Element distribution map of substrate region. (**a**) SEM micrograph; (**b**) Mo; (**c**) Si; (**d**) B.

Figure 3 shows the EDS plot of the substrate region, and it can be clearly seen that the surface element distribution of Mo-12Si-8.5B alloys made by discharge plasma sintering is relatively uniform. The yellow dotted line region is the α-Mo substrate. The α-Mo shows a discontinuous island distribution in the substrate, in which Mo_3_Si with a relatively high Si element content as well as Mo_5_SiB_2_ show a mesh-like distribution structure, which will have an adverse effect on the mechanical properties of the material.

### 3.2. Laser Remelting

**Figure 4:**
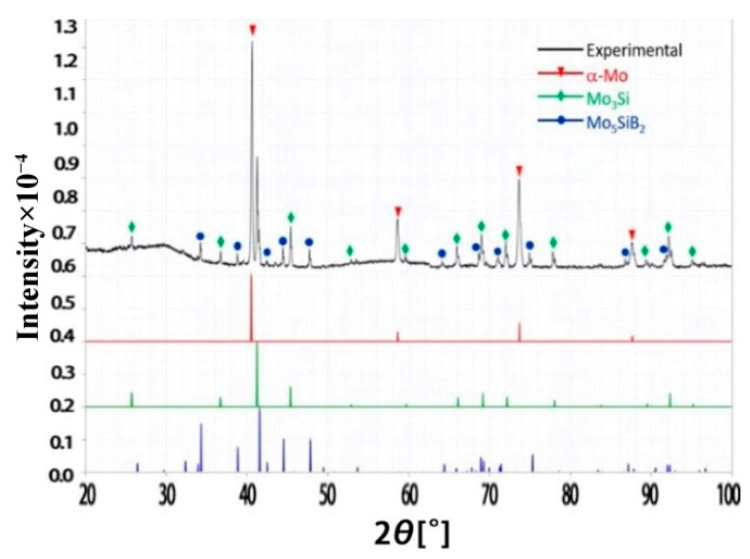
The XRD results of laser remelted Mo-12Si-8.5B alloy.

As shown in Figure 4, XRD results of the melt pool area on the surface of the laser remelted specimens showed that the processed specimens still consisted of three phases: α-Mo, Mo_3_Si and Mo_5_SiB_2_, indicating that the laser remelting process did not change the composition of the Mo-Si-B alloy surface phases. Therefore, the surface XRD results of the laser remelting treatment are not discussed separately.

#### 3.2.1. Laser Remelting Specimen Surface Microstructure Analysis

**Figure 5:**
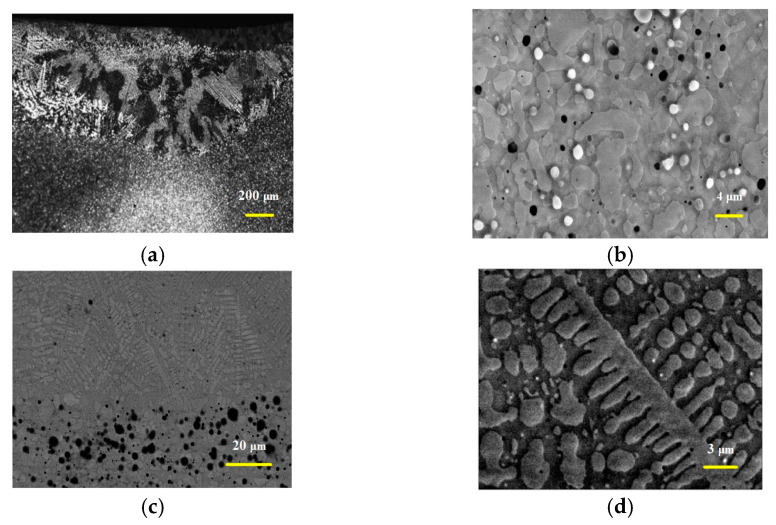
The surface microstructure of the Mo-Si-B alloy with laser remelting. (**a**) specimen surface morphology; (**b**)substrate area; (**c**) melt pool and substrate transition area (BSE); (**d**) melt pool area.

Figure 5 shows the SEM images of the cross-sectional microstructure of the Mo-Si-B alloy with the laser surface remelting process. Figure 5a shows that the boundary between the melt pool area and the substrate area on the surface of the specimen after the laser remelting process is clearly visible, and there is a large difference in its microstructure and morphology. Figure 5b shows the SEM image of the substrate region, which shows that the grain shape in this region is mainly irregular polygonal, and some holes are distributed on its surface, mainly on the phase boundary, which reach a size of about 1–2 µm. Figure 5c shows the backscattered electron imaging (BSE) images of the substrate and melt pool regions. It is obvious that the average length of the dendrites in the melt pool region is 70 µm. Only a few holes are distributed in the melt pool region and no cracks are found, while a large number of holes are distributed in the substrate region. The presence of a large number of holes reduces the density of the material and is detrimental to the mechanical properties of the alloy, but the laser remelting process can greatly reduce both the number and size of holes and improve the mechanical properties of the alloy. Figure 5d shows the microstructure of the upper-middle region of the melt pool, which shows that the dendritic arms are more developed in this region, indicating that the cooling rate of the upper-middle region of the melt pool has increased, and the microstructure is denser, which is beneficial to the improvement of mechanical properties.

#### 3.2.2. EDS Analysis in the Melt Pool

**Figure 6:**
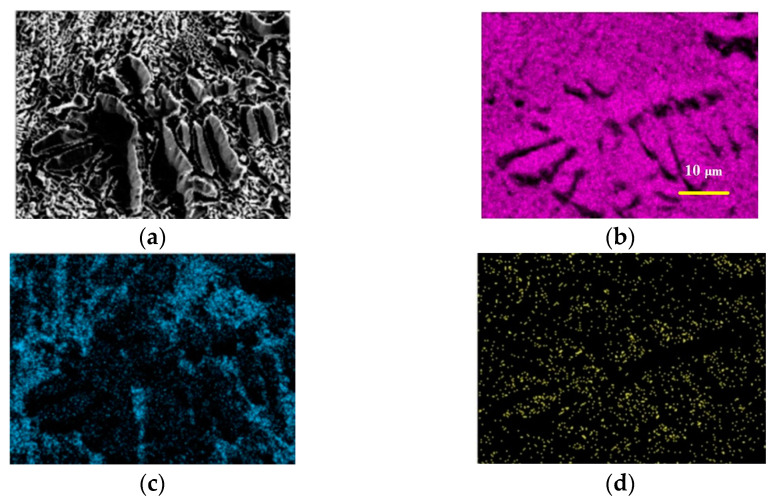
Element distribution map of melted area with laser remelting. (**a**) SEM micrograph; (**b**) Mo; (**c**) Si; (**d**) B.

Figure 6 shows the element distribution of the melt pool with laser remelting. It can be seen from Figure 6 that the Mo element content is higher in the dendrite region, while Si and B elements are higher in the interdendrite region. This indicates that segregation occurs during processing, which is related to the fast heating and cooling characteristics of the process. The α-Mo phase with a higher melting point is formed preferentially in the dendrite stem, while the Si- and B-rich Mo_3_Si and Mo_5_SiB_2_ phases with lower melting points are precipitated in the interdendrite region. The -Mo grains obtained by powder metallurgy are all polygonal in shape, while the melt pool region after remelting consists mainly of α-Mo (dendrite stem) and mixed Mo_3_Si and Mo_5_SiB_2_ (interdendrite). Since the melting points of the three are from high to low: α-Mo (2610 °C) > Mo_5_SiB_2_ (2200 °C) > Mo_3_Si (2022 °C), in the initial stage of solidification in the remelting zone, -Mo solidifies to generate dendritic stems, while Si and B are gradually crowded into the interdendritic region, followed by the formation of Mo_5_SiB_2_ and Mo_3_Si.

The experiments show that the surface morphology of the Mo-Si-B alloy specimens with the laser remelting process has been significantly changed. The original bright gray areas of the substrate are α-Mo phases with grain sizes of 2–5 µm, while these areas are surrounded by Mo_3_Si and Mo_5_SiB_2_ phases, and the dark gray areas are mainly Mo_3_Si phases. After the laser remelting, a large number of dendrites, mainly composed of α-Mo, appeared in the melt pool area, and the dendrite size ranged from 60 to 70 µm, while Si and B compounds with lower melting points were produced in the interdendrite area. Combined with the above experimental results, the laser remelting process can effectively change the substrate morphology to produce a structure with better properties than that produced by the powder sintering process.

### 3.3. Electron Beam Remelting

**Figure 7:**
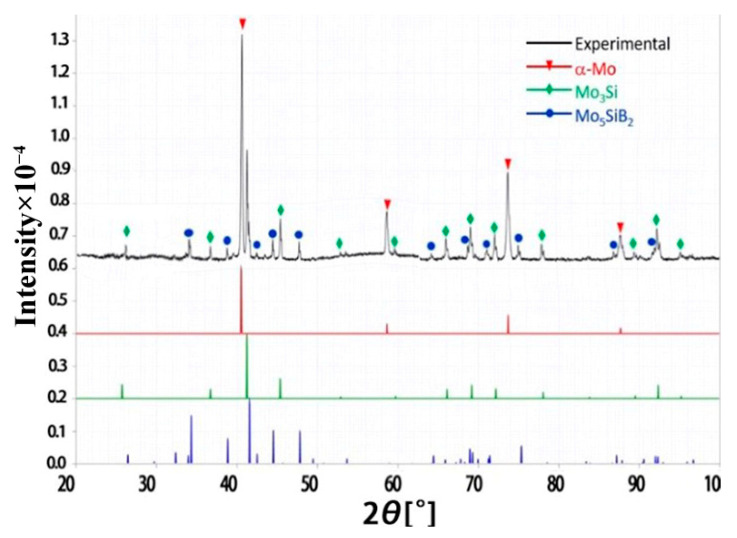
The XRD results of electron beam remelted Mo-12Si-8.5B alloy.

As shown in Figure 7, XRD analysis of the melt pool area on the surface of the processed specimens showed that they still consisted of three phases: α-Mo, Mo_3_Si and Mo_5_SiB_2_, indicating that the electron beam remelting process did not change the composition of the surface phases of the Mo-Si-B alloy. The XRD results indicate that the two high-energy beam surface remelting processes did not change the composition of the original material phases.

#### 3.3.1. SEM Micrograph

**Figure 8:**
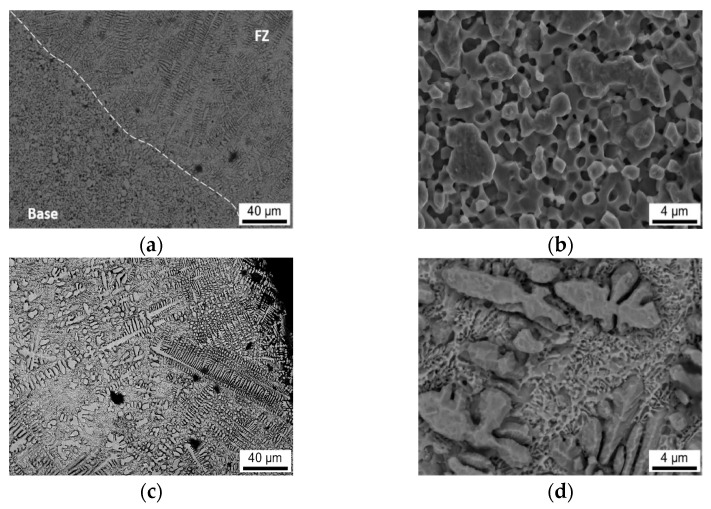
The surface microstructure of the Mo-Si-B alloy with electron beam remelting. (**a**) melt pool and substrate transition area; (**b**) substrate area; (**c**) specimen surface morphology; (**d**) interdendritic area of the melt pool.

Figure 8 shows the SEM images of the specimens processed by electron beam remelting. It can be seen from Figure 8a that, similar to the laser remelting treatment, the substrate region and the melt pool region have a clear demarcation line, and the substrate region has more fine holes, while the number of holes in the melt pool region is sharply reduced. Figure 8b shows the microstructure of the substrate region, and the grain structure is irregular polygonal in shape. Figure 8c is a picture of the melt pool area compared with the specimen after laser remelting; the dendrites in the melt pool area have a larger size with a length greater than 120 µm after electron beam remelting treatment. Figure 8d shows the interdendritic region of the upper-middle melt pool, which shows that the dendritic arms are less developed in this region, indicating that the cooling rate of the upper-middle region of the melt pool has decreased and the microstructure is less dense, which is harmful to the improvement of mechanical properties. Combined with the EDS analysis, the interdendritic region is mainly a Si-rich phase.

#### 3.3.2. EDS Analysis in the Melt Pool

**Figure 9:**
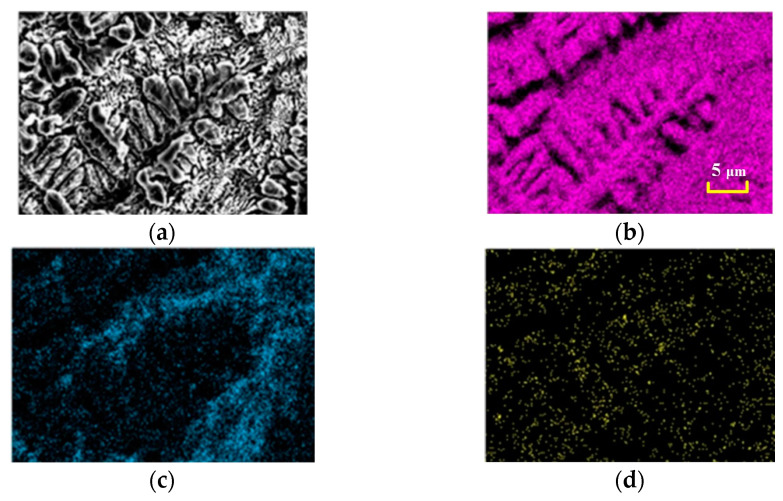
Element distribution map of melted area with electron beam remelting. (**a**) SEM micrograph; (**b**) Mo; (**c**) Si; (**d**) B.

Figure 9 shows the EDS diagram of the melt pool area of the specimen after the electron beam remelting process, and it can be clearly seen that, similar to the specimen after the laser remelting process, a large number of dendrites appear in the melt pool area, whose main composition is Mo. By combining Figure 9b,c, the content of B elements in the main dendrite area is more than that in the interdendrite area, which is related to the fast heating and cooling characteristics of the electron beam process, and the Si elements are mainly enriched in the interdendrite area.

### 3.4. Mechanical Property

**Figure 10:**
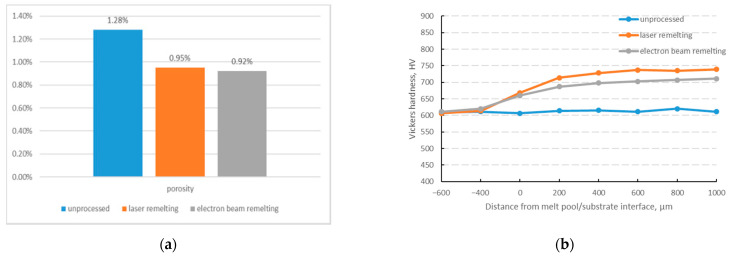
Mo-12Si-8.5B alloy with unprocessed, laser remelting and electron beam remelting. (**a**) porosity; (**b**) Vickers hardness.

Figure 10 shows the porosity and Vickers hardness of Mo-12Si-8.5B alloys with unprocessed laser and electron beam remelting. Specimens remelted by electron beam have the smallest porosity, as shown in Figure 10a. There is only a small difference in the Vickers hardness of the substrate before and after processing, as shown in Figure 10b. However, laser-remelted specimens have the highest Vickers hardness in the melt pool area.

From the above experimental results, it can be seen that both laser and electron beam remelting do not change the composition of the surface phase of the Mo-Si-B alloy. However, the microstructure of the Mo-Si-B alloy surface after remelting is different due to the various types of heat sources. The energy distribution of the electron beam is Gaussian distribution, which tends to cause the phenomenon of local overheating during processing, resulting in the formation of dendrites of larger sizes in the melt pool area and more serious segregation in the dendritic area. While the energy distribution of the laser is a flat-topped mode, which heats the material surface more uniformly, the specimens after laser remelting are more uniformly organized. Therefore, the use of laser remelting technology is beneficial to reduce the Mo-Si-B alloy surface porosity and dendrite segregation while improving its mechanical properties.

## 4. Conclusions

Laser and electron beam remelting with high temperature gradients and high cooling rates are new ways with great potential to achieve the three-dimensional forming of Mo-Si-B alloys. We have comparatively investigated the phase, microstructure, and element distribution of Mo-12Si-8.5B alloys with discharge plasma sintering, laser remelting and electron beam remelting. The main conclusions are as follows:

(1) Laser beam remelting and electron beam remelting on the surface of the Mo-12Si-8.5B alloy successfully reduce the number of holes in the melt pool area, which is beneficial to the mechanical properties of the alloy. The hole reduction is related to the characteristics of fast heat and fast cooling during the remelting processing.
(2) Laser remelting and electron beam remelting processing do not change the phase composition of the Mo-12Si-8.5B alloys but lead to element segregation in the dendrite region and continuous uniform distribution of α-Mo.
(3) The electron beam remelted specimens had the smallest number and size of holes. However, the laser remelted specimen had the smallest dendrite size of about 70 µm and the lightest dendrite segregation. In a comprehensive comparison, the laser is more suitable as the heat source for Mo-12Si-8.5B alloy processing.
(4) As the electron beam has the characteristics of high energy and fast temperature rise, it is easy to cause local overheating during processing. Moreover, its heat diffuses to the substrate rapidly, resulting in the formation of dendrites with a larger size in the melt pool area and significant element segregation in the dendrite area.

The research in this paper focuses on the microstructure of the remelting process. Future research will aim to establish an appropriate model based on temperature stress, mechanical properties and microstructure and to perform mechanical property experiments based on this paper, which will facilitate the ability to form complex parts of Mo-Si-B alloys and lay the foundations for the application Mo-Si-B alloys to aero-engine hot-end parts.

## Figures and Tables

**Table 1:** Processing groups.

**Group**	S1	S2	S3
**Process**	None	Laser Remelting	Electron Beam Remelting

## Data Availability

Not applicable.
